# Supplementary material for: Factors associated with quality of life among newly diagnosed acute ischemic stroke patients: a community-based case-control study
Source: PeerJ. 2024 Oct 16;12:e18266. doi: 10.7717/peerj.18266 (PMC11490225; doi:10.7717/peerj.18266)
Supplement: Supplemental Information 2 [file peerj-12-18266-s002.docx]

**Codebook**

**Group**

1 = Stroke patients

2 = Non-Stroke patients

**Sex**

1 = Male

2 = Female

**Birthyear**

Year of birth (Buddhist Era: B.E.)

**Age**

**Religion**

1 = Buddhism

2 = Christianity

3 = Islam

**Maritalstatus**

1 = Single

2 = Married

3 = Separated

4 = Widowed

5 = Divorced

**Educationlevel**

1 = Uneducated

2 = Primary School

3 = Junior High School

4 = Senior High School/ Vocational Certificate (Voc.Cert.)

5 = Diploma/High Vocational Certificate (Dip. / High Voc.Cert.)

6 = Bachelor’s Degree

7 = Master’s Degree

**Occupation**

1 = Agriculturist

2 = Employee/ Freelancer

3 = Business owner

4 = Government official/ Government employee

5 = Private employee/

Factory worker

6 = Do-nothing

7 = Housewife

8 = Other

**diagnosisYr**

Year of diagnosis with Diabetes Mellitus (DM) or Hypertension (HT)

**UD**

Underlying diseases

1 = No

2 = Yes (Please specify)

**HUD1**

Chronic kidney disease (CKD)

0 = No

1 = Yes

**HUD2**

Cardiovascular disease (CVD)

0 = No

1 = Yes

**HUD3**

Stroke

0 = No

1 = Yes

**HUD4**

Diabetes Mellitus (DM) or Hypertension (HT)

0 = No

1 = Yes

**HUD5**

Thalassemia

0 = No

1 = Yes

**HUD6**

Other Underlying diseases

**Histparalysis**

Family members (such as parents and siblings) have been diagnosed with paralysis

1 = No

2 = Yes (Please specify)

**HistCVD**

Family members (such as parents and siblings) have been diagnosed with Cardiovascular disease (CVD)

1 = No

2 = Yes (Please specify)

**Weight**

**Height**

**BMI**

Body Mass Index (BMI)

**Smoking**

1 = Never smoked

2 = Stopped smoking (before diagnosed with DM or HT)

3 = Stopped smoking (after diagnosed with DM or HT)

4 = Stopped smoking (for public)

5 = Still smoking

**Alcohol**

1 = Never drink alcohol

2 = Stopped drinking (before diagnosed with DM or HT)

3 = Stopped drinking (after diagnosed with DM or HT)

4 = Stopped drinking (for public)

5 = Still drinking

**Circumwaist**

Waist Circumference

**QL1-26**

quality of life questionnaires

1 = Not at all

2 = A little

3 = A moderate amount

4 = Very much

5 = Extremely
